# Serum proteome alterations during conventional and extracorporeal resuscitation in pigs

**DOI:** 10.1186/s12967-022-03441-4

**Published:** 2022-05-23

**Authors:** Patrick Bernhard, Berit Amelie Bretthauer, Sam Joé Brixius, Hannah Bügener, Johannes Elias Groh, Christian Scherer, Domagoj Damjanovic, Jörg Haberstroh, Georg Trummer, Christoph Benk, Friedhelm Beyersdorf, Oliver Schilling, Jan-Steffen Pooth

**Affiliations:** 1 Institute for Surgical Pathology, Medical Center-University of Freiburg, Faculty of Medicine, University of Freiburg, Freiburg, Germany; 2 Spemann Graduate School of Biology and Medicine (SGBM), University of Freiburg, Freiburg, Germany; 3 Faculty of Biology, University of Freiburg, Freiburg, Germany; 4 Department of Cardiovascular Surgery, University Heart Center Freiburg, University Medical Center Freiburg, Faculty of Medicine, University of Freiburg, Hugstetter Str. 55, 79106 Freiburg, Germany; 5 Department of Experimental Surgery, Center for Experimental Models and Transgenic Service, University Medical Center Freiburg, Faculty of Medicine, University of Freiburg, Freiburg, Germany

**Keywords:** Cardiopulmonary resuscitation, Proteome, Extracorporeal membrane oxygenation, Extracorporeal cardiopulmonary resuscitation, Ischemia reperfusion injury

## Abstract

**Background:**

Only a small number of patients survive an out-of-hospital cardiac arrest (CA) and can be discharged from hospital alive with a large percentage of these patients retaining neurological impairments. In recent years, extracorporeal cardiopulmonary resuscitation (ECPR) has emerged as a beneficial strategy to optimize cardiac arrest treatment. However, ECPR is still associated with various complications. To reduce these problems, a profound understanding of the underlying mechanisms is required. This study aims to investigate the effects of CA, conventional cardiopulmonary resuscitation (CPR) and ECPR using a whole-body reperfusion protocol (controlled and automated reperfusion of the whole body—CARL) on the serum proteome profiles in a pig model of refractory CA.

**Methods:**

N = 7 pigs underwent 5 min of untreated CA followed by 30 min CPR and 120 min perfusion with CARL. Blood samples for proteomic analysis were drawn at baseline, after CPR and at the end of the CARL period. Following albumin-depletion, proteomic analysis was performed using liquid chromatography–tandem mass spectrometry.

**Results:**

N = 21 serum samples were measured resulting in the identification and quantification of 308–360 proteins per sample and 388 unique proteins in total. The three serum proteome profiles at the investigated time points clustered individually and segregated almost completely when considering a 90% confidence interval. Differential expression analysis showed significant abundance changes in 27 proteins between baseline and after CPR and in 9 proteins after CARL compared to CPR. Significant findings were further validated through a co-abundance cluster analysis corroborating the observed abundance changes.

**Conclusions:**

The presented data highlight the impact of systemic ischemia and reperfusion on the entire serum proteome during resuscitation with a special focus on changes regarding haemolysis, coagulation, inflammation, and cell-death processes. Generally, the observed changes contribute to post-ischemic complications. Better understanding of the underlying mechanisms during CA and resuscitation may help to limit these complications and improve therapeutic options.

**Supplementary Information:**

The online version contains supplementary material available at 10.1186/s12967-022-03441-4.

## Background

In Europe, depending on the region, 28 to 244 out of 100,000 people suffer from out-of-hospital cardiac arrest (OHCA) each year [1]. Despite continuous efforts to optimize resuscitation care and infrastructure, on average only 8% of all patients survive an OHCA and can be discharged from hospital alive [2]. Moreover, a large percentage of these patients show persistent neurological impairments even in the case of successful resuscitation with return of spontaneous circulation (ROSC) [3, 4]. Therefore, the continuous improvement of treatment strategies for cardiac arrest (CA) patients remains a declared goal of the global resuscitation community [5].

In recent years, the clinical use of devices for extracorporeal circulation during resuscitation (ECPR) has proven to be beneficial in selected patients [6, 7]. Consequently, ECPR has already been implemented in international resuscitation guidelines [8, 9]. However, the use of extracorporeal circulation devices is associated with various complications, such as increased hemolysis, increased bleeding propensity and activation of inflammatory pathways [10–12]. Each of these complications on its own has the potential to promote cell death and to negatively affect the outcome of a prolonged resuscitation [13].

Successful limitation and treatment of these complications require a profound understanding of the underlying mechanisms. Most of the current research in the field of resuscitation is focused on proteomic changes after ROSC in order to identify markers for neurological outcome [14–16]. Proteomic changes induced by either conventional cardiopulmonary resuscitation (CPR) or ECPR remain rather neglected and unclear.

Considering the multiple factors that can contribute to a poor outcome after CA, it seems obvious that CA treatment requires an individualized, targeted reperfusion to limit cell death and subsequent complications. Therefore, a whole-body reperfusion protocol (controlled automated reperfusion of the whole body—CARL) was established over the last decade and has proven to allow for complete cerebral recovery in selected patients even after prolonged periods of CA and CPR [17].

To improve therapeutical concepts in CPR, we aimed at providing fundamental, unbiased insights into proteome alterations during CA and subsequent CPR and CARL. The objective of this study was to assess the effects of guideline-based advanced life support (ALS) CPR followed by CARL on the serum proteome during the first hours of resuscitation in a pig model of refractory CA.

## Methods

The experiments were authorized by the local animal welfare committee (Regierungspräsidium Freiburg, G-15/148). All experiments were conducted in accordance with the rules and regulations of the German animal protection law and the animal care guidelines of the European Community (2010/63/EU).

### Anesthesia and surgical procedure

**Fig. 1:**
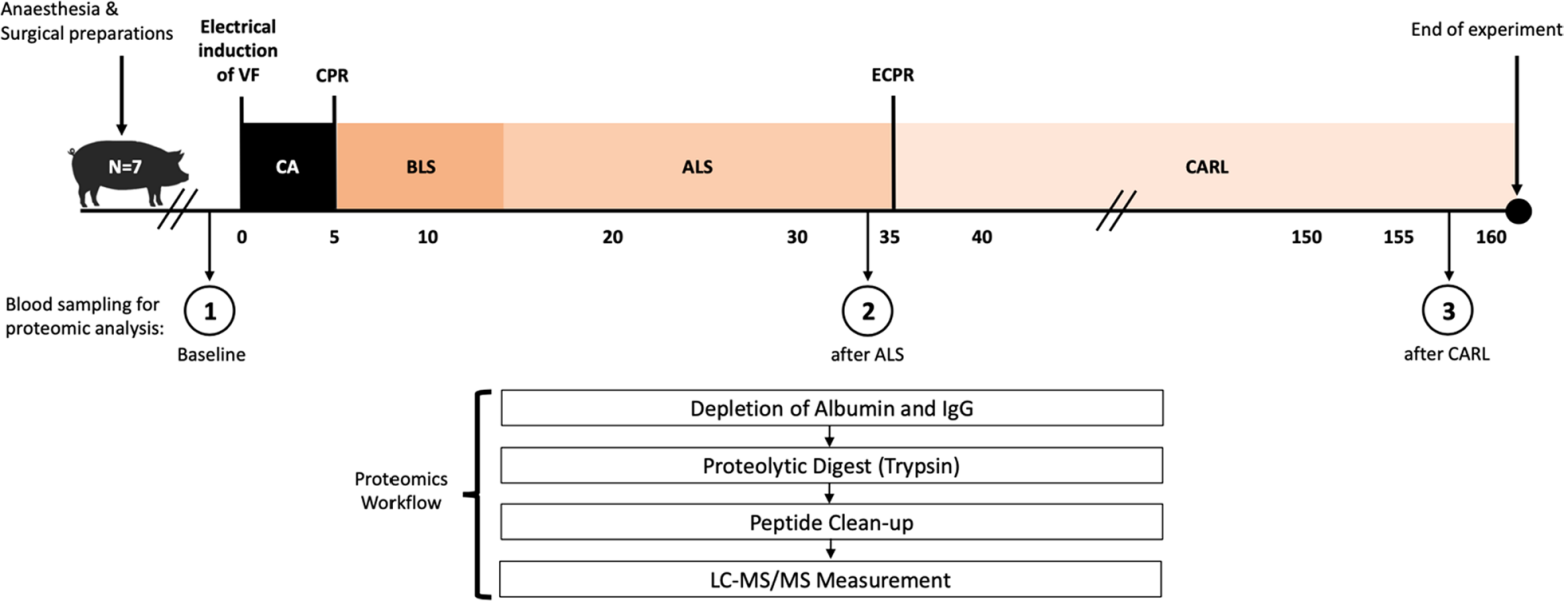
Schematic experimental design and proteomics workflow. Pigs underwent a successive series of treatments including cardiac arrest (CA), cardiopulmonary resuscitation (CPR), basic life support (BLS), advanced life support (ALS) and extracorporeal cardiopulmonary resuscitation (ECPR) using CARL (controlled automated reperfusion of the whole body). Blood sampling was performed at the three marked time points including “Baseline” (time point 1), “after ALS” (time point 2) and “after CARL” (time point 3). Subsequent proteomic sample processing involved depletion of albumin and immunoglobulin G (IgG), proteolytic digest and peptide clean-up before samples were subjected to liquid chromatography–tandem mass spectrometry (LC–MS/MS) measurement. VF, ventricular fibrillation

Figure 1 shows the experimental protocol. The experimental protocol was designed using real-life process times in OHCA. Seven male Landrace hybrid pigs of 50–70 kg bodyweight (kgBW) were premedicated with an intramuscular injection of midazolam and ketamine in the animal facility. After establishing total intravenous anesthesia with propofol and vecuronium, the pigs underwent endotracheal intubation and were transported to the operation room. Anesthesia was maintained with isoflurane, fentanyl and vecuronium. Volume-controlled ventilation settings were chosen to normalize pH, partial pressure of arterial oxygen (paO_2_) and carbon dioxide (paCO_2_). Ringer’s solution was used as a primary fluid substitute, and antibiotic prophylaxis was provided with ceftriaxone. Cannulation of the right common carotid artery was achieved via a right paratracheal surgical dissection to obtain continuous arterial blood pressure monitoring and interval blood sampling for arterial blood gas analysis. The right internal jugular vein was cannulated to obtain blood samples for proteomic analysis. A nasopharyngeal probe was inserted for continuous temperature management. Due to pulmonary embolism, one animal could not be weaned of the extracorporeal circulation at the end of the experiment. Therefore, the remaining three animals received heparin (300 IU kgBW^−1^) before the induction of CA.

### Cardiac arrest

In case of an OHCA, bystanders witness the collapse, and call emergency medical services (EMS), which leads to the dispatch of emergency ambulances (Fig. 1). After obtaining all baseline measurements, the pigs underwent five minutes of normothermic CA induced by ventricular fibrillation (VF) using electrical stimulation via pacing electrodes of a Swan-Ganz catheter. During these five minutes, no resuscitation, ventilation, or therapy of any kind was administered. For veno-arterial extracorporeal circulation, the right external jugular vein was cannulated for venous drainage with a 22–24 F cannula and the right femoral artery with a 14–16 F cannula for arterial inflow. These cannulas were flushed with 5.000 IU heparin each and stayed clamped during the CA and CPR period.

### Basic and advanced life support

After five minutes of untreated CA, CPR was initiated. Since the average EMS response time in Germany is 13–15 min, basic life support (BLS) was provided for 8 min following the International Liaison Committee on Resuscitation (ILCOR) recommendations. Chest compressions were performed using a mechanical compression device (Corpuls cpr, GS Elektromedizinische Geräte G. Stemple GmbH, Kaufering, Germany) with a frequency of 100 min^−1^ and a compression depth of 4–6 cm. Ventilation was conducted with a self-inflating bag.

Following 5 min of CA and 8 min of BLS, EMS arrive on scene and start ALS (Fig. 1). Cardiac rhythm assessments, ventilations and applications of adrenaline were conducted according to international guidelines [8, 9]. Unlike ILCOR recommendation, defibrillations and antiarrhythmic drug therapy were omitted over the whole ALS period to standardize the simulation of refractory VF and avoid ROSC. ALS was provided for 22 min.

### Extracorporeal cardiopulmonary resuscitation

Extracorporeal cardiopulmonary resuscitation is only recommended in prolonged resuscitation attempts when conventional CPR is failing. A German national consensus statement labels a resuscitation attempt as prolonged after 20 min of CPR [18]. Following 5 min of CA and a total of 30 min of ALS and BLS with refractory VF, all pigs received CARL therapy for two hours. As common in clinical practice, all animals also received 5000 IU of heparin at the initiation of CARL. The CARL® system (Resuscitec GmbH, Freiburg, Germany) was used as extracorporeal circulation device with specific reperfusion targets like pulsatile blood flow maintained between 5 and 6 L min^−1^, hypothermia up to 32 °C in the first hours of reperfusion and a mean arterial blood pressure (MAP) between 80 and 120 mmHg [19].

### Proteomics sample preparation and analysis

Blood samples for proteomic analysis were drawn at baseline, at the end of ALS and after 120 min of CARL. Samples were centrifuged at 2000*g* for 15 min at 10 °C. Serum was transferred to cryo-tubes, aliquoted and immediately stored at – 80 °C until further analysis.

For mass spectrometry (MS) sample preparation, all serum samples were depleted for albumin and IgG using the ProteoExtractTM Albumin/IgG Removal Kit (Merck, Darmstadt, Germany) according to manufacturer´s instructions with 35 µL serum. Protein concentration of each depleted serum sample was determined by using the BCA assay (Thermo Fisher Scientific, Waltham, USA) before subjecting 100 µg of serum proteome to in-solution tryptic digestion. Acid-labile surfactant (AcLS, sodium 3-[(2-methyl-2-undecyl-1,3-dioxolan-4-yl)methoxy]-1-propanesulfonate) was added to a final concentration of 0.1% (v/v) and 0.1 M HEPES (4-(2-hydroxyethyl)-1-piperazineethanesulfonic acid, pH 7.5), before boiling the samples for 10 min at 95 °C. Cystine reduction was performed using 5 mM dithiothreitol (30 min, 37 °C) and subsequent alkylation using 15 mM iodoacetamide (30 min, room temperature, in the dark). Protein pre-digestion was performed by adding 2 µg Lysyl Endopeptidase (Wako Chemicals, Neuss, Germany) (protease:protein ratio 1:50) and incubating for 2 h at 42 °C, before adding 4 µg trypsin (Worthington Biochemical Corporation, Lakewood, USA) (protease:protein ratio 1:25) and further incubate samples for 17 h at 37 °C. Digestion was stopped by acidification to pH < 2 through the addition of trifluoroacetic acid (TFA, Thermo Fisher Scientific, Waltham, USA) to a final concentration of 2% (v/v). AcLS was precipitated by incubating the acidified samples for 30 min at 37 °C and centrifuging for 10 min at 20,000*g* at room temperature. Clear supernatant was used for peptide desalting/clean-up using iST C18 mixed phase cartridges (PreOmics, Martinsried, Germany) according to manufacturer´s instructions. After determining the peptide concentration via the BCA assay, eluates were vacuum dried and stored at − 80 °C until analysis.

For MS measurement, vacuum dried peptides were resolubilized in 0.1% (v/v) formic acid to a final concentration of 0.2 µg/µL, sonicated for 5 min and centrifuged at 20,000*g* for 10 min before transferring the supernatant to the measurement tube. 1 µg of each sample, together with 100 fmol of indexed retention time (iRT) peptides, were analyzed using a nanoflow liquid chromatography (LC) system, Easy-nLC 1000 (Thermo Fisher Scientific, Waltham, USA) equipped with a trapping column (Fused Silica Capillary; 3 cm length, 100 μm inner diameter, VICI Jour, Schenkon, Switzerland) and an analytical column (Self-Pack PicoFrit Column; 40 cm length, 75 μm inner diameter, New Objective, Woburn, MA) both in-house packed[20] with C18 particles (Dr. Maisch, ReproSil-Pur 120 C18-AQ; 3 μm C18 particle size, 120 Å pore size). Samples were trapped at 200 bars with 100% buffer A (0.1% v/v formic acid) and separated using a reverse phase C18 column with the analytical column temperature set at 60 °C and at a flow rate of 300 nL/min. A multistep gradient of 5% to 65% buffer B (80% v/v acetonitrile, 0.1% v/v formic acid, 1% v/v monoethylene glycol) in buffer A was used for separation, followed by washing (100% B) and reconditioning of the column to 5% B (see Additional file 3: Table S1 for detailed gradient overview).

The Easy-nLC 1000 system was coupled online to an Orbitrap Elite or Q-Exactive plus (Thermo Fisher Scientific, Waltham, USA) mass spectrometer respectively via a Nanospray Flex Ionsource (Thermo Fisher Scientific, Waltham, USA). The analytical column contains an integrated uncoated pre-cut emitter (Silica Tip, 10 μm tip inner diameter, New Objective, Woburn, MA) and a voltage of approximately 2.7 kV were applied for electrospray ionization. The mass spectrometer was operated in data dependent acquisition mode and each MS scan was followed by a maximum of 16 MS/MS scans (Top16 method). The mass range from 300 to 2000 m/z (mass-to-charge ratio) was analyzed. MS1 resolution was set to 70,000, automatic gain control (AGC) to 3e6 and maximum injection time was set to 50 ms. MS2 resolution was set to 17,500, AGC to 1e5 and maximum injection time to 80 ms.

### Statistical analysis

MaxQuant (V1.6.17.0) software was used for data analysis [21]. Peptide identification was performed using the Andromeda search engine [22] with a pig proteome database containing reviewed Uniprot sequences without isoforms downloaded from Uniprot on 23^rd^ March 2020 (22,161 entries). Decoys for the database search were generated in MaxQuant by using the revert function. The precursor mass tolerance for the initial search was 20 ppm and for the main search 4.5 ppm, whereas the fragment mass tolerance was 20 ppm. Tryptic cleavage specificity with 2 missed cleavages was applied while setting a minimal peptide length of seven amino acids. Carbamidomethyl at cysteines was the only fixed modification, whereas setting methionine oxidation and protein N-terminal acetylation as variable modifications allowing a maximum of 5 modifications per peptide. The false discovery rate for peptides and proteins was set to 0.01. Label-free quantitation (LFQ) was performed using the MaxLFQ algorithm and enabled Match-between-Run function.

The MaxQuant output was further processed in R (V 4.0.3) with RStudio (V 2021.09.1) as an integrated development environment. After filtering for unique peptides, protein summarization of LFQ intensities (Tukey´s median polish), log2-transformation and median centering was performed by using the MSstats package (V 3.22.1) [23]. Biognosys iRT-peptide entries were removed from the protein list. First, to investigate the effect of CPR and ECPR on the overall serum proteome profiles, we performed a partial least squares discriminant analysis (PLSDA) between the three time points. PLSDA was performed by using the mixOmics package (V 6.16.0). Second, we performed a multigroup limma approach for pairwise statistical testing to identify differentially expressed proteins between the considered time points in our experiments regarding the applied methods of resuscitation. Differential expression analysis was performed using the limma package (V 3.48.3). Proteins, which displayed a statistically significant abundance change (adjusted p-value ≤ 0.05), were assigned to one of the four predefined categories: hemolysis, coagulation, inflammation, and cell death. As a third and independent analysis, we evaluated the abundance behavior of all identified proteins over time by performing a co-abundance cluster analysis using the publicly available Clust algorithm (V 1.10.10), which assigns each identified protein to a certain abundance-course with a confidence interval of 95%. Clust algorithm [24] was executed in Python (V 3.8.9) using Z-score normalization prior to k-means clustering with seed number k = 10 and tightness weight t = 1.0. Gene Ontology annotation was manually performed using the publicly available STRING database[25] and UniProt database[26].

All statistical analyses to describe the animal experiments were conducted using R statistical software (version 4.0.3). To control for the assumption of normally distributed data, a Shapiro–Wilk test was performed. Normally distributed data were expressed as mean ± standard deviation and otherwise as median with interquartile range.

## Results

Seven male pigs with an average weight of 58.7 ± 9.4 kg underwent the experimental setup. Table 1 shows the intraoperative parameters. Despite sufficient thoracic compressions, which resulted in an end-tidal CO_2_ of 25.5 [23.75; 33] mmHg and a mean MAP of 31 [26.5; 41] mmHg, the pigs reached an arterial pH of 7.06 ± 0.13 and a mean lactate concentration of 8.7 ± 1.9 mmol L^−1^ after 30 min of CPR. After initiation of CARL, all animals were supported with a mean extracorporeal blood flow of 6.1 ± 1.0 L min^−1^ and a corresponding MAP of 72 ± 15 mmHg. While ventricular fibrillation was persistent in all animals until the end of the CPR period, all animals showed an organized cardiac rhythm after 120 min of CARL. However, three animals presented intermittent returns to unorganized rhythms during CARL and required defibrillations (Table 1).

**Table 1:** Animal data

	Before CA ("Baseline")	30 min CPR ("after ALS")	120 min CARL ("after CARL")
Hemoglobin [g L^−1^]	8.9 ± 0.6	12.8 ± 1.1	8.4 ± 0.7
MAP [mmHg]	82 ± 5	31 [26.5; 41]	72 ± 15
End-tidal CO_2_ [mmHg]	39 ± 2	25.5 [23.75; 33]	26 ± 15
paO_2_ [mmHg]	94.4 ± 17.7	66.7 ± 30.8	96.1 ± 19.6
paCO_2_ [mmHg]	38.0 ± 3.8	68.0 ± 24.8	41.8 ± 4.1
Arterial pH	7.47 ± 0.03	7.06 ± 0.13	7.30 ± 0.03
Lactate [mmol L^−1^]	1.4 ± 0.5	8.7 ± 1.9	12.8 ± 2.9
Defibrillations	0 [0; 0]	0 [0; 0]	3 [0; 6]
Total amount of norepinephrine [mg]	0 [0; 0]	0 [0; 0]	6.1 ± 3.2
Total amount of epinephrine [mg]	0 [0; 0]	5 [5; 5]	5 [5; 5]
Total amount of infused volume [ml]	0 [0; 0]	215 ± 35	1665 ± 388
Mean extracorporeal blood flow [L min^−1^]	n.a	n.a	6.1 ± 1.0

21 serum samples were processed and measured as described above, resulting in the identification and quantification of 308–360 proteins per sample and 388 unique proteins in total.

### PLSDA

**Fig. 2:**
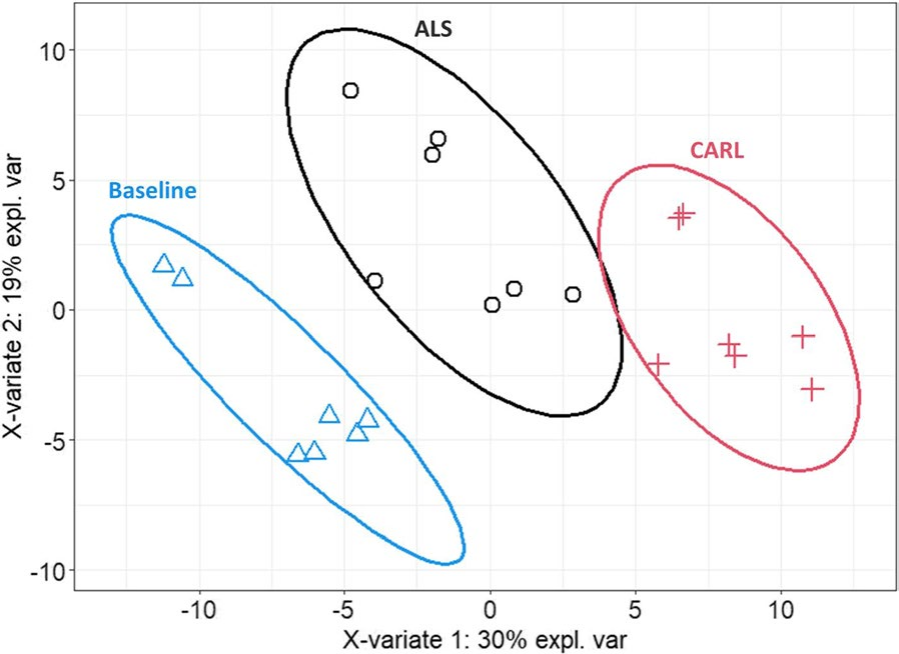
Partial least squares discriminating analysis (PLSDA). Protein profiles of each sample, together with the corresponding time point annotation (Baseline, ALS, CARL) were submitted to PLSDA analysis. Ellipses represent an 90% confidence interval

Figure 2 shows the results of the PLSDA analysis. It is evident that the serum proteome profiles of the three time points cluster individually and almost completely segregate from each other when considering a 90% confidence interval. While the overall serum proteome profile after ALS showed a clear segregation compared to the baseline profile, this divergence to the baseline profile seemed to be even higher for the serum proteome profile after CARL.

### Differential expression analysis

**Fig. 3:**
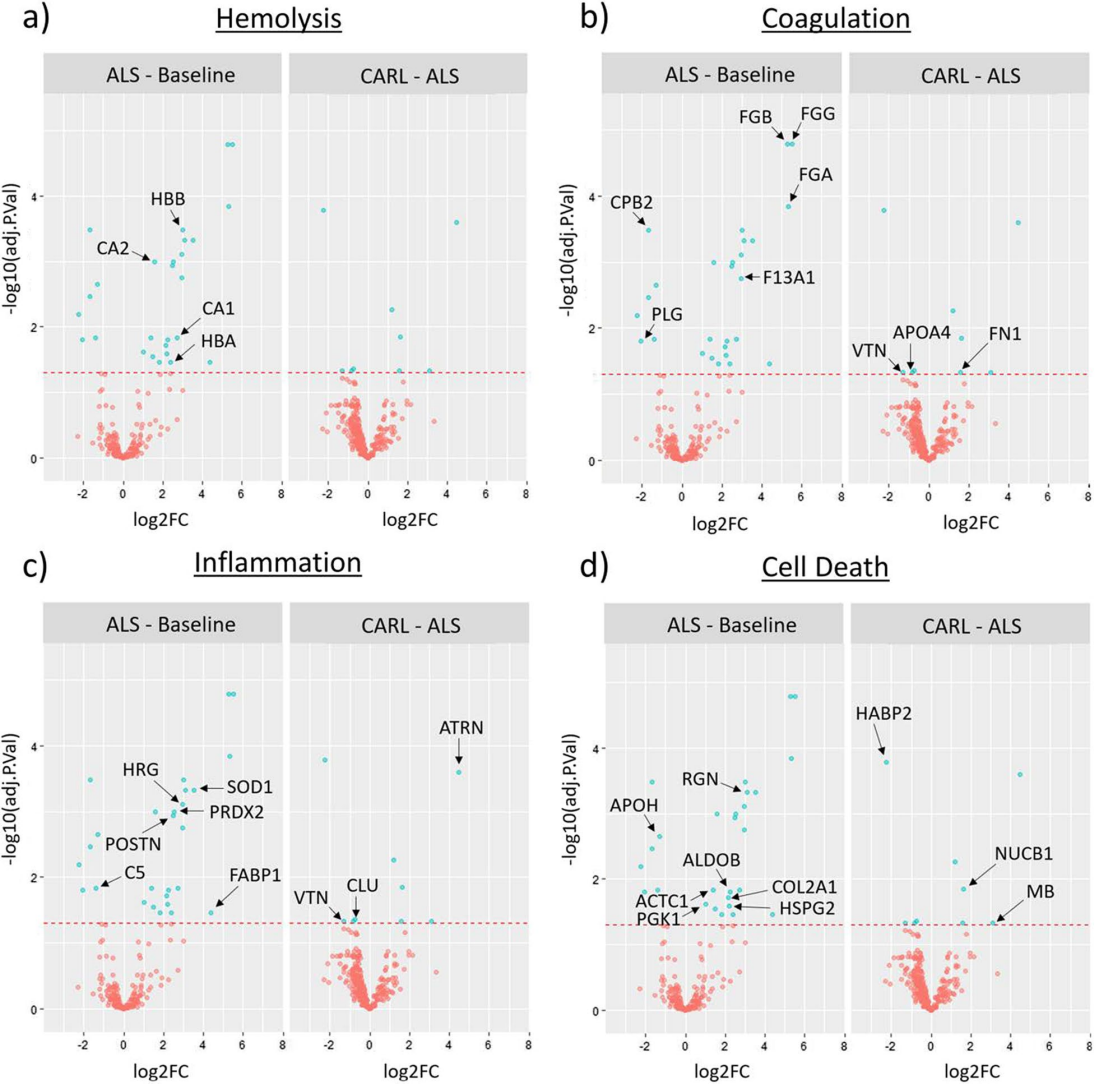
Differential expression analysis using pairwise multigroup limma. The volcano plots **a**–**d** show the results of the same pairwise differential expression analysis between “ALS-Baseline” and “CARL-ALS”. The log2 fold changes (log2FC) are plotted on the x-axis and corresponding adjusted p-values in − log10 scale is shown on the y-axis. The applied adjusted p-value cut-off was set to 0.05 (1.3 in – log10 scale), which is depicted as dashed horizontal line. Each plot highlights significantly up- or downregulated proteins (blue dots) of the respective biological process: **a** Hemolysis, **b** Coagulation, **c** Inflammation, **d** Cell Death. Hereby, a log2FC > 0 corresponds to an upregulation in the first-mentioned condition. Protein datapoints are labelled with corresponding gene names (black arrows) retrieved from UniProt database (Additional file 4: Table S2)

A total of 36 significantly differentially expressed proteins were identified (27 for ALS vs Baseline; 9 for CARL vs ALS) (see also Additional file 4: Table S2). These significantly differentially expressed proteins were assigned to one of the four predefined categories as appropriate (Fig. 3).

For hemolysis-related proteins, the differential expression analysis revealed a significant increase in abundance after ALS for the alpha and beta subunit of hemoglobin (HBA, HBB) as well as for carboanhydrase 1 (CA1) and carboanhydrase 2 (CA2) (Fig. 3a).

Proteins involved in the coagulation process showed a significant increase after ALS for factor XIII (F13A1) and fibrinogen alpha, beta and gamma chain (FGA, FGB, FGG), whereas plasminogen (PLG) and carboxypeptidase B2 (CPB2) revealed a significant decrease in abundance during that period (Fig. 3b). Furthermore, the analysis of coagulation-related proteins showed a significantly increased abundance after CARL for fibronectin (FN1), whereas apolipoprotein A-IV (APOA4) and vitronectin (VTN) revealed a significant decrease during CARL.

For proteins involved in inflammation, we identified a significantly increased abundance after ALS for periostin (POSTN), peroxiredoxin-2 (PRDX2), superoxide dismutase 1 (SOD1), fatty acid-binding protein (FABP1) and histidine rich glycoprotein (HRG), whereas complement 5a anaphylatoxin (C5) showed a significant decrease in abundance (Fig. 3c). During CARL, the inflammation-related protein attractin (ATRN) revealed a significant increase, whereas the protein clusterin (CLU) showed a significantly decreased abundance. Besides the coagulation-process, the protein vitronectin (VTN) could also be assigned to the inflammation-process.

Cell-death related proteins showed a significantly increased abundance after ALS for the proteins phosphoglyceratkinase (PGK1), actin alpha cardiac muscle 1 (ACTC1), fructose-bisphosphat aldolase (ALDOB), collagen type II (COL2A1), regucalcin (RGN) and heparansulfate proteoglycan 2 (HSPG2), whereas apolipoprotein H (APOH) revealed a reduced abundance (Fig. 3d). During CARL, nucleobinin-1 (NUCB1) and myoglobin (MB) revealed a significantly increased abundance, whereas hyaluronan-binding protein 2 (HABP2) showed a significantly decreased abundance.

### Co-abundance clustering

**Fig. 4:**
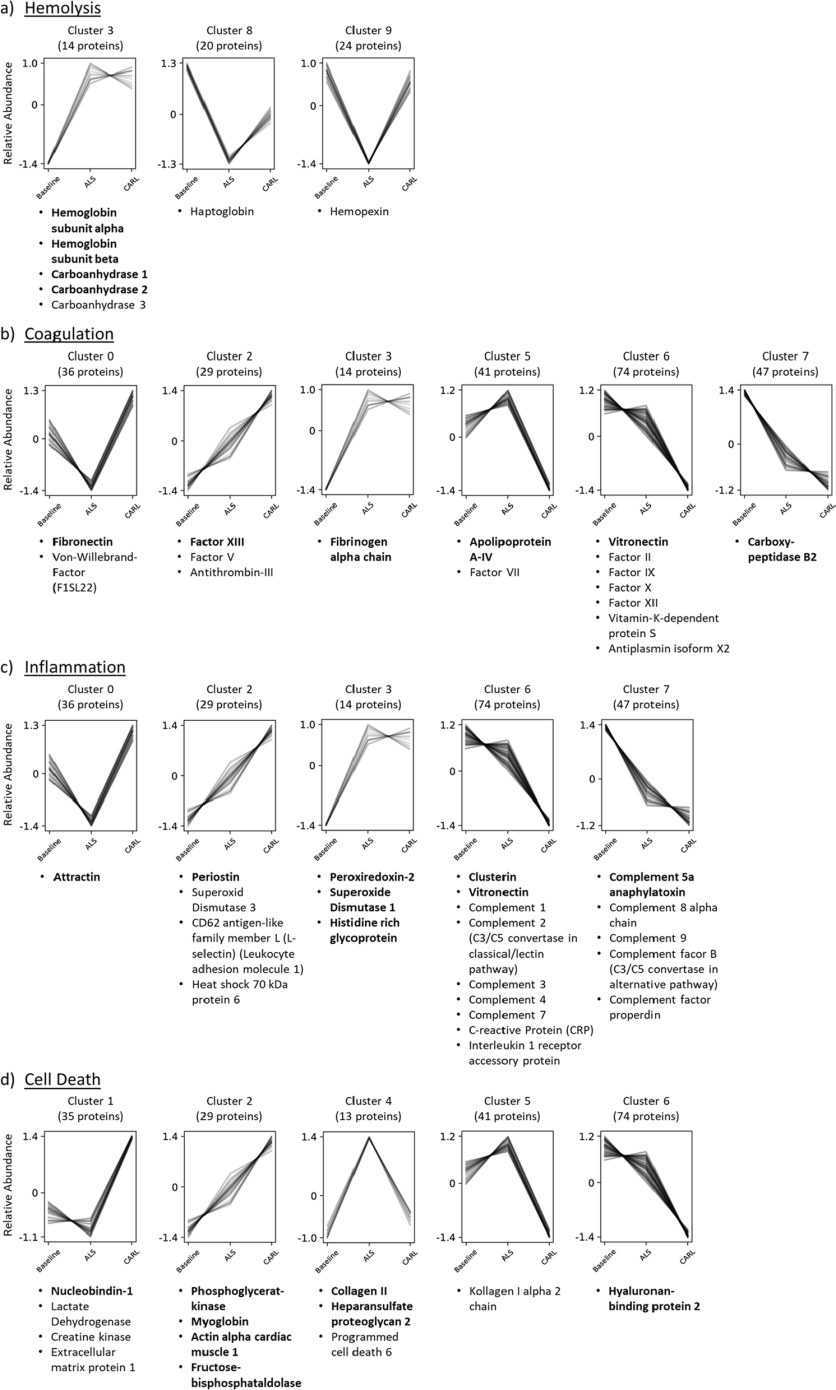
Protein hits and their assigned co-abundance cluster for each considered biological process. Depending on the measured intensities at different time points of the experiment (Baseline, ALS, CARL), the identified proteins were assigned to different co-abundance cluster with a confidence interval of 95%. Cluster assignment was performed by using the Clust algorithm and assigned proteins were manually annotated to one of the respective biological processes: **a** hemolysis, **b** coagulation, **c** inflammation, **d** cell death. Each line represents an individual protein, while y-axis illustrates the relative abundance change after Z-score normalization. Bold protein names represent proteins that already showed a significant abundance change (adjusted p-value ≤ 0.05) during to the previous differential expression analysis (Fig. 3) for at least one of the considered comparisons. The number of assigned proteins per cluster is shown above each graph

The co-abundance clusters showed ten distinct abundance-courses with a total of 333 assigned proteins (Additional file 1: Fig. S1). To validate the results of the previous differential expression analysis, we identified the significant protein hits in the resulting co-abundance clusters. This was possible for 26 of the differentially expressed proteins and corroborated the previously observed abundance changes (bold entries in Fig. 4 and Additional file 2: Fig. S2).

Besides the differentially expressed proteins, we identified further proteins of each biological process in the co-abundance clusters (Fig. 4). Although these proteins did not show a statistically significant abundance change during the limma analysis of the two considered comparisons, they were assigned to a specific abundance behavior.

For example, carboanhydrase 3 was found in the same cluster as the significantly differentially expressed carboanhydrase 1 and 2, and which thereby further supports a uniform behavior of all observed isoforms (Fig. 4a). Furthermore, the hemoglobin-binding proteins haptoglobin and hemopexin showed—as expected—an inverse abundance behavior in comparison to hemoglobin after ALS, and thereby further corroborated the previously observed effects on hemoglobin (Fig. 4a).

It should be noted that in some cases, several representatives of the same biological process were assigned to the identical co-abundance cluster, illustrating a collective abundance behavior during the experiment. This accumulation of proteins with shared function can for example be observed for several coagulation factors (factor II, IX, X, XII and VII), which show only a slight abundance change after ALS, whereas the abundance was drastically reduced during CARL (Fig. 4b). However, other coagulation factors like factor V and XIII rather show a continuous increase in abundance over the whole experiment.

Likewise, several components of the complement system (C1, C2, C3, C4 and C7) showed the same abundance behavior as described above, whereas further complement components (C5, C8, C9 and complement factor B) revealed a different kinetic with a steeper decrease after ALS and a flattening of the decrease during CARL (Fig. 4c). Furthermore, extracellular superoxide dismutase 3 shows a continuous increase in abundance over the whole experiment and thereby contrasts with the cytoplasmic superoxide dismutase 1, which revealed a flattened or even stagnating increase during CARL treatment.

## Discussion

It is generally acknowledged that whole-body ischemia reperfusion injury (IRI) occurs after CA and during subsequent CPR [27, 28]. Since it is possible, with consistent treatment and limitation of IRI via targeted extracorporeal perfusion, to push the limits of neurological resuscitability [29] and increase survival, especially neurological survival, after CA [17], gaining a better understanding of the underlying mechanisms is urgently needed to improve therapeutical concepts in CPR.

Multiple pathological processes such as autoantibody and complement activation, no-reflow-phenomena, vascular leakage and cell death programs are triggered by uncontrolled reperfusion after ischemia [29, 30]. As the use of extracorporeal circulation devices can trigger similar complications [10–12] and in prolonged resuscitation encounters an already damaged organism, we aimed to provide a closer examination of these processes.

These changes, which occur in the context of IRI, are interdependent, but can be divided into four categories based on their pathophysiology: hemolysis, coagulation, inflammation, and cell death.

### Hemolysis

In resuscitation, the depletion of energy carriers leads to an electrolyte shift [31]. If this disturbance of osmolality is exacerbated by uncontrolled reperfusion (i.e., restoration of sub-physiological flows through chest compressions), fluids shift to extravascular compartments [31] and lead to an intravascular rise in high-molecular proteins like hemoglobin (Table 1). Consequently, resulting in the swelling and bursting of erythrocytes. Since hemolysis is also a common complication in extracorporeal membrane oxygenation due to high shear stress mainly caused by the pumps [32], the question arose, whether hemolysis is further exacerbated during CARL treatment.

In this set of experiments, we observed all significant increases in abundance of hemolysis associated proteins (hemoglobin subunit alpha and beta, carboanhydrase 1 and 2) during conventional CPR (Fig. 3a). The observed clusters associated with these proteins further support this finding, indicating that no additional hemolysis was triggered during CARL (cluster 3, 8 and 9, Fig. 4a). Interestingly, haptoglobin und hemopexin even showed the tendency to recover during CARL treatment. Based on these results, we conclude that CA and conventional CPR, rather than CARL, represent the main trigger for hemolysis in a prolonged resuscitation.

### Coagulation

As for changes in coagulation, some authors have already postulated that alterations in coagulation factors and fibrinolysis can be observed during conventional CPR that fulfil the definition of a disseminated intravascular coagulation [33]. In a recent review, Gando and Wada identified damage-associated-molecular-patterns, inflammatory cytokines and adrenaline as triggers for platelet activation, thrombin and fibrin formation, insufficient anticoagulation pathways and increased fibrinolysis [33]. We were also able to observe significant changes after CA and conventional CPR (e.g., fibrinogen alpha chain, factor XIII, carboxypeptidase B2), which support the afore mentioned thesis of a disseminated intravascular coagulation in prolonged conventional CPR (Fig. 3b). This was further strengthened by the abundance clusters of the individual coagulation factors (Fig. 4b). Here, the use of CARL seemed to intensify the impairment of the coagulation system. Fibronectin was significantly increased and apolipoprotein A-IV significantly decreased at the end of the CARL period, contributing to an even more dysregulated coagulation [34, 35]. Vitronectin also significantly decreased, contributing to a profibrinolytic state during CARL [36]. These findings were accompanied by the observation of a pulmonary embolism in one animal at the end of the observation period, which emphasizes the clinical significance of the observed changes.

Additionally, an increased von Willebrand factor concentration was detected during CARL (cluster 0, Fig. 4b). Although an increased release from the endothelium is to be expected in reperfusion, the von Willebrand factor may have been functionally inactive due to degradation by the extracorporeal circuit [37]. It is known that the use of extracorporeal circulation devices leads to a functional loss of von Willebrand factor and the consecutive occurrence of an acquired von Willebrand syndrome [38, 39].

In general, our findings highlight the importance of anticoagulation in a reperfusion strategy for ECPR. In particular, the timing, type and duration of an optimal anticoagulation strategy need further investigation.

### Inflammation

Regarding inflammation, the consequences of ischemia and reperfusion are comparable to mechanisms activated by invading microorganisms. Hence, IRI can be defined as a sterile inflammation, which is characterized by the formation of reactive oxygen species (ROS), accumulation of inflammatory cells and activation of the complement system [30].

ROS, which are a main contributor to IRI, originate from hypoxia activated neutrophils and intramitochondrial decoupling of the respiratory chain [29, 33]. Once formed, ROS are responsible for exacerbating IRI-mediated intracellular calcium overload, which leads to mitochondrial dysfunction and activation of cell death pathways [29]. Our study revealed a significant increase of intracellular antioxidative proteins in prolonged resuscitation (i.e., peroxiredoxin-2, superoxide dismutase 1), indicating that CA and conventional CPR are triggers of significant ROS formation (Fig. 3c) [40, 41]. As depicted in the associated abundance clusters, proteins associated with intracellular ROS formation remained elevated during CARL, while superoxide dismutase 3, a marker for extracellular ROS formation, even showed an increasing trend (cluster 2, Fig. 4c). Consequently, limiting oxidative stress appears to be under-represented in current resuscitation strategies and could be an important factor in post-resuscitation care. As some studies have already shown a protective effect of the endogenous antioxidant SOD1 upon global ischemia in rats [42] as well as an exacerbated cell damage upon SOD1 deficiency in mice [43], the induction of antioxidant enzymes via activation of NRF2 signaling as well as the application of SOD-catalase-mimics could represent promising treatment targets for antioxidant therapy, some of which are already under evaluation in clinical trials [44].

Accumulation of inflammatory cells is mediated by various signaling proteins. This includes attractin, which is found on activated T cells and mediates their interaction with macrophages [45]. Attractin showed a decreased abundance after CA and conventional CPR (cluster 0, Fig. 4c) with a consecutive increase after CARL, which proved to be statistically significant (Fig. 3c). This change in attractin, in the context of the corresponding abundance cluster, could hint at a recovery of the dysregulated inflammatory system after restoration of energy sources during CARL. Its course needs to be interpreted in the context of the largest group of identified proteins of the inflammatory system, which belonged to the complement system.

The complement system is a central system for immune surveillance and plays a key role in host homeostasis, inflammation and the defense against pathogens [46]. During ischemia and reperfusion the complement system seems to fuel inflammation and the recruitment of immune cells through complement-mediated recognition of damaged cells and anaphylatoxin release [30]. Additionally, it is also involved in regenerative processes (e.g., hematopoietic stem cell engraftment, bone growth, and angiogenesis) [47]. By showing a significant decrease after ALS, complement 5a anaphylatoxin appeared to be excessively activated during CA and conventional CPR (Fig. 3c). Interestingly, complement 8 alpha chain, complement 9 and C3/C5 convertase from the alternative pathway showed the same abundance behavior after CA and conventional CPR (cluster 7, Fig. 4c), which indicates activation of the complement system via the alternative pathway and consequently the formation of the membrane attack complex (MAC). Complement 1–4 and C3/C5 convertase of the classical pathway together with clusterin and vitronectin were associated with a different abundance cluster (cluster 6, Fig. 4c). Their abundance behavior showed a significant decrease during CARL, indicating an excessive activation after exposure to the extracorporeal circuit. Clusterin and vitronectin represent inhibitors of the MAC [48]. Therefore, their decrease during CARL could correspond to a disinhibition of the MAC and also signal a beginning normalization of the dysregulated inflammatory response. However, longer observation periods are needed to validate such a conclusion.

### Cell death

The ultimate aim and individual measure of success of any resuscitation therapy is, of course, the limitation of cellular injury. Ischemia and reperfusion activate multiple programs of cell-death (i.e., necrosis, apoptosis and autophagy-associated cell-death) [49]. Cell-death after ischemia and reperfusion not only contributes to tissue damage and organ dysfunction, but it is also highly immunostimulatory. While this has been long known for necrosis, recent studies also show a similar response in apoptosis [30].

In our study, we were able to observe significant increases in markers of cellular injury after prolonged resuscitation, as expected (e.g., actin alpha cardiac muscle 1, phosphoglyceratkinase and fructose-bisphosphat aldolase A) (Fig. 3d). Nucleobindin-1, which is a ubiquitous intracellular protein and therefore a reliable marker for global tissue injury [50], showed a clear increase after CARL in association with lactate dehydrogenase (LDH) and creatin kinase (CK) (cluster 1, Fig. 4d). When looking at these abundance clusters one could conclude that cellular injury shows a linear increase over the course of the experiment (cluster 2, Fig. 4d) or is even exacerbated during CARL (cluster 1, Fig. 4d). We would argue that these observations are proof of the late hit of a prolonged resuscitation with insufficient flows as provided by conventional CPR. A large number of energy-depleted cells are already in an irreversible cell death process and cannot be saved, since the restoration of sufficient flows cannot reverse existing injuries [19, 29]. So, these abundance clusters point to the great, unrealized potential of organ protection in conventional CPR.

Furthermore, it has already been shown that post cardiac arrest patients display increased concentrations of glycosaminoglycans as a sign of endothelial injury after CA [51]. Since endothelial injury contributes to the capillary leak syndrome and organ dysfunction [52], it is associated with unfavourable outcome after CA [53, 54]. In our study, heparansulfate proteoglycan 2 together with collagen II showed a significant increase at the end of the conventional CPR period (Fig. 3d). While heparansulfate proteoglycan 2 is part of the endothelial glycocalyx [51], collagen II represents a protein of the extracellular matrix [55]. Both proteins showed a return to baseline values after initiation of CARL (Cluster 4, Fig. 4d), thus pointing to a recovery of endothelial function and absence of further injuries to the endothelium during CARL.

### Limitations of this study

Due to the experimental design and the time points of serum sampling, it is not possible to differentiate the effects of CA and CPR, as we did not collect a sample before starting CPR. Consequently, the observed effects at the “after ALS” time point are additive effects of CA and CPR. Furthermore, no defibrillations were performed, and no amiodarone was administered during CPR to avoid premature ROSC. The authors are not aware of any evidence pointing to proteome changes regarding defibrillations or drug administration during resuscitation. Nevertheless, the authors acknowledge that both might represent confounders regarding short-term proteome alterations (for example higher parameters of cell death if repeated defibrillations were applied). Due to the observation of thrombotic events, heparin was administered prior to the induction of CA in three animals (identified in the online repository). Although we did not observe clear differences in the overall serum profile of animals regarding the administration of additional heparin (Fig. 2), we did not investigate potential changes that could be attributed to the administration of heparin. Additionally, the observation period of the experiment comprises 160 min and thereby is too short to observe gene regulatory effects on protein level. Thereby, we only observe acute and direct effects on the already present serum proteins. To also consider gene regulatory effects on protein level, one would have to include an additional sample after several days.

## Conclusions

The presented study offers fundamental insights into the effects of CA, conventional CPR and CARL on the serum proteome of pigs. Our data demonstrated significant changes in proteins associated with biological processes such as hemolysis, coagulation, inflammation, and cell-death. Most importantly, we observed normalization of hemolysis parameters during CARL, signs of a disseminated intravascular coagulation and ROS formation after prolonged conventional CPR and endothelial damage after conventional CPR with a recovery during CARL. Since oxidative stress and ROS formation play an important role in exacerbating cellular injury and cell death, we suggest that more attention should be paid to antioxidant therapy in post-resuscitation care. Further unbiased insights into the pathophysiology of refractory CA are urgently needed to develop therapeutical concepts, which interrupt the vicious circle of ischemia and reperfusion in CPR and contribute to an improvement in outcome after CA.

## Supplementary Information

**Additional file 1: Figure S1.** Co-abundance cluster analysis of identified serum proteins. This figure shows the ten identified co-abundance clusters.

**Additional file 2: Figure S2.** Co-abundance clusters of significantly differentially expressed proteins. This figure displays all proteins, which were found to be significantly differentially expressed in the multigroup limma analysis, in their identified co-abundance clusters.

**Additional file 3: Table S1.** Applied chromatographic gradient for peptide separation prior to mass spectrometric measurement. The table describes the proportion of Buffer B in Buffer A over time for peptide separation prior to mass spectrometric measurement.

**Additional file 4: Table S2.** Proteins revealing a significant abundance change for multigroup limma analysis. The table displays all proteins which revealed a significant abundance change in the statistical analysis. It includes adjusted p-values and UniProt IDs.

## Data Availability

All mass spectrometry proteomics datasets used and/or analysed during this study are available online at the MassIVE repository (http://massive.ucsd.edu/; dataset identifier: MSV000088595; Reviewer account details: Username: “MSV000088595_reviewer”, Password: “PigSerumeCPR"). All datasets regarding the animal experiments are available from the corresponding author upon reasonable request.

## Abbreviations

AcLS: Acid-labile surfactant
ALS: Advanced life support
BLS: Basic life support
CA: Cardiac arrest
CARL: Controlled automated reperfusion of the whole body
CPR: Cardiopulmonary resuscitation
ECPR: Extracorporeal cardiopulmonary resuscitation
EMS: Emergency medical services
expl. var.: Explained variance
iRT: Indexed retention time
LC: Liquid chromatography
LFQ: Label-free quantification
log2FC: Log2 fold change
MS: Mass spectrometry
n.a.: Not applicable
OHCA: Out-of-hospital cardiac arrest
paO_2_: Partial pressure of arterial oxygen
paCO_2_: Partial pressure of arterial carbon dioxide
PLSDA: Partial least squares discriminant analysis
ROS: Reactive oxygen species
ROSC: Return of spontaneous circulation
RT: Room temperature
TFA: Trifluoroacetic acid
VF: Ventricular fibrillation
