# Supplementary material for: Serum proteome alterations during conventional and extracorporeal resuscitation in pigs
Source: J Transl Med. 2022 May 23;20:238. doi: 10.1186/s12967-022-03441-4 (PMC9125930; doi:10.1186/s12967-022-03441-4)

**Figure S1:** **Co-Abundance Cluster Analysis of identified serum proteins.** Abundance-courses of identified proteins over the three time points (Baseline, after ALS, after eCPR) were assigned to ten distinct co-abundance clusters. The clustering is based on k-means clustering and assigns the proteins with a confidence interval of 95 %. Each line represents the relative abundance change (y-axis) of one individual protein over time. The number of assigned proteins per cluster is shown above each cluster.


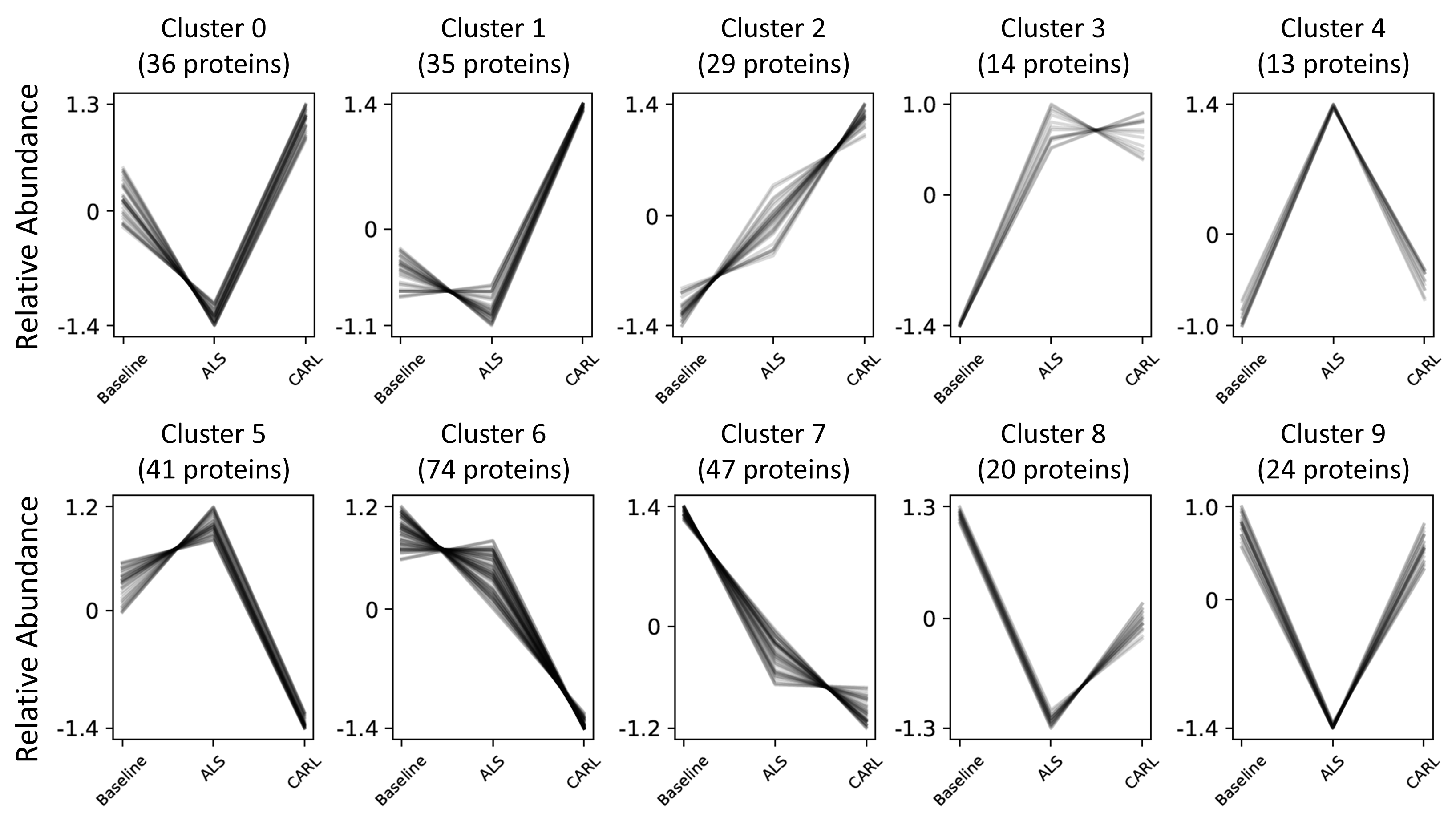

Supplement: Supplementary file 1 — Additional file 1: Figure S1. Co-abundance cluster analysis of identified serum proteins. This figure shows the ten identified co-abundance clusters. [file 12967_2022_3441_MOESM1_ESM.docx]
