# Supplementary material for: Serum proteome alterations during conventional and extracorporeal resuscitation in pigs
Source: J Transl Med. 2022 May 23;20:238. doi: 10.1186/s12967-022-03441-4 (PMC9125930; doi:10.1186/s12967-022-03441-4)

**Figure S2:** **Co-Abundance Clusters of significantly differentially expressed proteins.** Depending on the measured intensities at different time points of the experiment (Baseline, After ALS, After eCPR), the identified proteins, which were previously found to be significantly differentially expressed (adjusted p-value ≤ 0.05), were assigned to different co-abundance cluster with a confidence interval of 95 %. Cluster assignment was performed by using the Clust algorithm and assigned proteins were manually annotated to one of the respective biological processes: a) Hemolysis, b) Coagulation, c) Inflammation, d) Cell Death. Each line represents an individual protein, while y-axis illustrates the relative abundance change after Z-score normalization. The number of assigned proteins per cluster is shown above each graph. The table below each cluster show in which pairwise comparison the respective protein revealed a significant abundance change during previous differential expression analysis (see Figure 3).


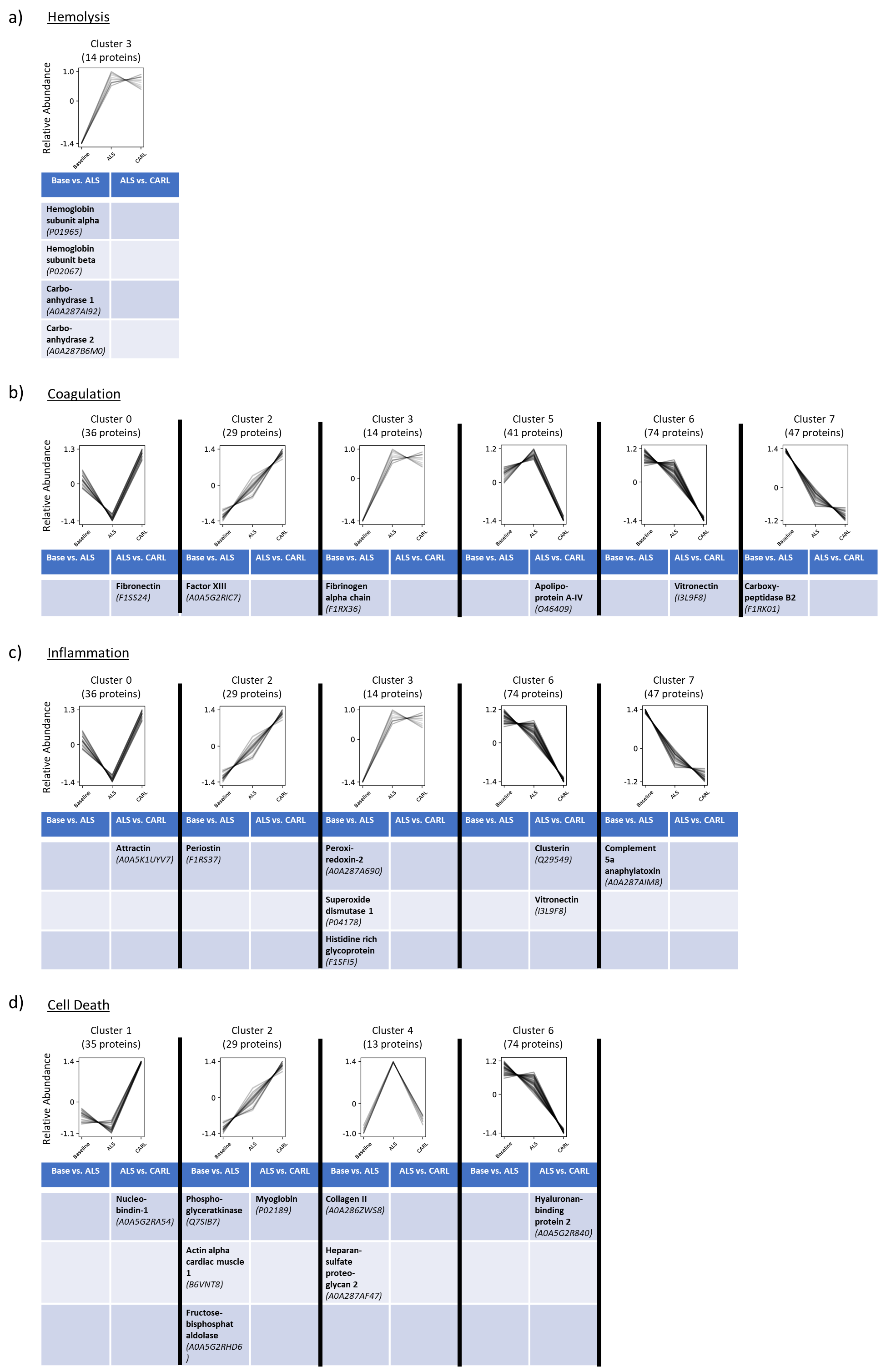

Supplement: Supplementary file 2 — Additional file 2: Figure S2. Co-abundance clusters of significantly differentially expressed proteins. This figure displays all proteins, which were found to be significantly differentially expressed in the multigroup limma analysis, in their identified co-abundance clusters. [file 12967_2022_3441_MOESM2_ESM.docx]
