# Supplementary material for: Serum proteome alterations during conventional and extracorporeal resuscitation in pigs
Source: J Transl Med. 2022 May 23;20:238. doi: 10.1186/s12967-022-03441-4 (PMC9125930; doi:10.1186/s12967-022-03441-4)
Supplement: Supplementary file 3 — Additional file 3: Table S1. Applied chromatographic gradient for peptide separation prior to mass spectrometric measurement. The table describes the proportion of Buffer B in Buffer A over time for peptide separation prior to mass spectrometric measurement. [file 12967_2022_3441_MOESM3_ESM.docx]

**Table S1: Applied chromatographic gradient for peptide separation prior to mass spectrometric measurement.** The table describes the proportion of Buffer B (80 % v/v Acetonitrile, 0.1 % v/v Formic acid, 1 % v/v Monoethylene glycol) in Buffer A (0.1 % v/v Formic acid) over time, while maintaining a constant flow rate.

| Time [min] | Duration [min] | Flow [nL/min] | % B |
| --- | --- | --- | --- |
| 0 | Start | 300 | 5 |
| 3 | 3 | 300 | 8 |
| 93 | 90 | 300 | 45 |
| 111 | 18 | 300 | 65 |
| 112 | 1 | 300 | 100 |
| 120 | 8 | 300 | 100 |
| 121 | 1 | 300 | 5 |
