# Supplementary material for: Serum proteome alterations during conventional and extracorporeal resuscitation in pigs
Source: J Transl Med. 2022 May 23;20:238. doi: 10.1186/s12967-022-03441-4 (PMC9125930; doi:10.1186/s12967-022-03441-4)
Supplement: Supplementary file 4 — Additional file 4: Table S2. Proteins revealing a significant abundance change for multigroup limma analysis. The table displays all proteins which revealed a significant abundance change in the statistical analysis. It includes adjusted p-values and UniProt IDs. [file 12967_2022_3441_MOESM4_ESM.docx]

**Table S2: Proteins revealing a significant abundance change for multigroup limma analysis.** All identified proteins were subjected to multigroup limma analysis. Listed proteins revealed a significant abundance change with an adjusted p-value ≤ 0.05 for the stated comparisons. Protein name and respective Gene name were retrieved from the UniProt database based on the specified UniProt identifier.

| Compar-ison | Protein Name | UniProt ID | Gene Name | Log2 Fold Change (log2FC) | Adjusted p-Value (adj.P.Val) |
| --- | --- | --- | --- | --- | --- |
| ALS vs Baseline  (Upregulated in ALS) | | | | | |
|  | Fibrinogen gamma chain | I3LJW2 | FGG | 5,5301 | < 0,0001 |
|  | Fibrinogen alpha chain (Fibrinopeptide A) | F1RX36 | FGA | 5,3137 | 0,0001 |
|  | Fibrinogen beta chain (Fibrinopeptide B) | F1RX37 | FGB | 5,2702 | < 0,0001 |
|  | Fatty acid-binding protein | P49924 | FABP1 | 4,3895 | 0,0341 |
|  | Superoxide dismutase 1 | P04178 | SOD1 | 3,5103 | 0,0005 |
|  | Regucalcin | Q06AA3 | RGN SMP30 | 3,0791 | 0,0005 |
|  | Haemoglobin subunit beta | P02067 | HBB | 3,0266 | 0,0003 |
|  | Histidine rich glycoprotein | F1SFI5 | HRG | 2,9482 | 0,0008 |
|  | Coagulation factor XIII A chain | A0A5G2RIC7 | F13A1 | 2,9426 | 0,0018 |
|  | Carbonic anhydrase 1 | A0A287AI92 | CA1 | 2,7332 | 0,0145 |
|  | Peroxiredoxin-2 | A0A287A690 | PRDX2 | 2,5345 | 0,0010 |
|  | Periostin | F1RS37 | POSTN | 2,4611 | 0,0012 |
|  | Haemoglobin subunit alpha | P01965 | HBA | 2,4004 | 0,0341 |
|  | Fructose-bisphosphate aldolase | A0A5G2RHD6 | ALDOB | 2,2486 | 0,0156 |
|  | Heparan sulfate proteoglycan 2 | A0A287AF47 | HSPG2 | 2,1771 | 0,0253 |
|  | Collagen type II alpha 1 chain | A0A286ZWS8 | COL2A1 | 2,1408 | 0,0190 |
|  | Secretoglobin family 1A member 1 (Uteroglobin) | F1RPX3 | SCGB1A1 | 1,8398 | 0,0341 |
|  | Carbonic anhydrase 2 | A0A287B6M0 | CA2 | 1,5902 | 0,0010 |
|  | Albumin | P08835 | ALB | 1,4758 | 0,0281 |
|  | Actin alpha cardiac muscle 1 | B6VNT8 | ACTC1 | 1,4122 | 0,0145 |
|  | Phosphoglycerate kinase 1 | Q7SIB7 | PGK1 | 1,0318 | 0,0242 |
| ALS vs Baseline  (Downregulated in ALS) | | | | | |
|  | BPTI/Kunitz inhibitor domain-containing protein | A0A5G2QN91 | LOC100158011 | -2,2441 | 0,0065 |
|  | Plasminogen | P06867 | PLG | -2,0552 | 0,0154 |
|  | Carboxypeptidase B2 | F1RK01 | CPB2 | -1,6909 | 0,0003 |
|  | Sulfhydryl oxidase | A0A5G2QXX3 | QSOX1 | -1,6837 | 0,0034 |
|  | Complement C5a anaphylatoxin | A0A287AIM8 | C5 | -1,3874 | 0,0145 |
|  | Apolipoprotein H | A0A286ZFW3 | APOH | -1,3044 | 0,0022 |
| CARL vs ALS  (Upregulated in CARL) | | | | | |
|  | Attractin | A0A5K1UYV7 | ATRN | 4,4694 | 0,0003 |
|  | Myoglobin | P02189 | MB | 3,0983 | 0,0466 |
|  | Nucleobindin-1 | A0A5G2RA54 | NUCB1 | 1,6395 | 0,0143 |
|  | Fibronectin | F1SS24 | FN1 | 1,5728 | 0,0466 |
|  | Apolipoprotein B-100 | A0A287AG13 | APOB | 1,2046 | 0,0055 |
| CARL vs ALS  (Downregulated in CARL) | | | | | |
|  | Hyaluronan-binding protein 2 | A0A5G2R840 | HABP2 | -2,2536 | 0,0002 |
|  | Vitronectin | I3L9F8 | VTN | -1,3301 | 0,0466 |
|  | Apolipoprotein A-IV | O46409 | APOA4 | -0,8370 | 0,0466 |
|  | Clusterin (CP40) | Q29549 | CLU | -0,7282 | 0,0441 |
